# Clinical significance and prognostic value of small nucleolar RNA SNORA38 in breast cancer

**DOI:** 10.3389/fonc.2022.930024

**Published:** 2022-09-09

**Authors:** Jian Song, Ang Zheng, Shan Li, Wenrong Zhang, Meilin Zhang, Xingzhe Li, Feng Jin, Ziyao Ji

**Affiliations:** ^1^ Department of Breast Surgery, The First Hospital of China Medical University, Shenyang, China; ^2^ Department of Ultrasound, The First Hospital of China Medical University, Shenyang, China

**Keywords:** SNORA38, breast cancer, prognosis, clinical implication, stemness

## Abstract

**Background:**

Breast cancer is the most common malignant tumor among women worldwide, and breast cancer stem cells (BCSCs) are believed to be the source of tumorigenesis. New findings suggest that small nucleolar RNAs (snoRNAs) play a significant role in tumor development.

**Methods:**

The Cancer Genome Atlas (TCGA) and Kaplan–Meier survival analysis were used to demonstrate expression and survival of SNORA38 signature. *In situ* hybridization (ISH) and immunohistochemical (IHC) were conducted to analyze the correlation between SNORA38 and stemness biomarker in 77 BC samples. Gene Set Enrichment Analysis (GSEA) was performed to investigate the mechanisms related to SNORA38 expression in BC. Real-time qPCR was employed to evaluate the expression of SNORA38 in breast cancer cell lines.

**Results:**

In the public database and patients’ biopsies, SNORA38 was significantly up-regulated in breast cancer. Furthermore, the expression of SNORA38 was significantly correlated with tumor size, lymph node metastasis, and TNM stage, among which tumor size was an independent factor for SNORA38 expression. Higher SNORA38 expression was associated with shorter overall survival (OS). Meanwhile, SNORA38 was positively associated with the stem cell marker OCT-4, which suggested that SNORA38 might be related to breast cancer stemness.

**Conclusions:**

SNORA38 is an important carcinogenic snoRNA in breast cancer and might be a prognostic biomarker for breast cancer.

## Introduction

Worldwide, breast cancer is one of the most common malignant tumors and has become the leading cause of cancer death among women (1). There were an estimated 1.7 million cases of breast cancer in women in 2012 (2), climbing to 2.1 million in 2018, accounting for almost a quarter of all cancers in women (3). Although “de-escalation” based on proven therapies has resulted in better outcomes for appropriate patients, it still requires more valuable evidence and rigorous judgment for breast cancer patients. Therefore, screening prognostic markers function is an effective method (4).

Heterogeneous tumor cell clusters exist in solid tumors, among which a particular subtype called cancer stem cells (CSCs) are characterized by self-renewal and pluripotency (5, 6). Breast cancer stem cells (BCSCs) are considered the root of differentiation, invasion, metastasis, drug resistance, and breast cancer recurrence. They have shown promising prospects in cancer therapy in recent years (7–9). Non-coding RNAs (ncRNAs), which do not code for proteins within the genome, has received particular interest in molecular research due to their new regulatory role in human health and disease. Small nucleolar RNAs (snoRNAs), about 60–300 nucleotides in length, are one of the most diverse non-coding RNAs and a kind of non-coding RNA widely present in the nucleoli of eukaryotic cells (10, 11). Previously, some snoRNAs were once thought to have a single function and limited role in pre-RNA processing. However, recent findings suggest that some snoRNAs are also involved in various physiological processes such as cell proliferation, differentiation, epigenetic inheritance, and regulation (12). The first report highlighting the pathological importance of snoRNAs showed that H5sn2 (a H/ACA box snoRNA) was significantly down-regulated in meningiomas (13). Abnormal regulation of snoRNAs has been confirmed as a tumor suppressor gene or oncogene in various cancers, including lung cancer, colorectal cancer, and other cancers (14–18). Expression analysis of clinical samples and cell lines indicates that snoRNAs are differentially expressed and may play a diagnostic and prognostic role in breast cancer (19–21).

However, the new role and potential mechanisms of SNORA38 in breast cancer remain unclear. We analyzed the expression of SNORA38 in 77 breast cancer samples and reported the relationship between SNORA38 and clinical-pathological, prognosis for the first time. In the present study, the Cancer Genome Atlas–breast cancer (TCGA-BRCA), Kaplan–Meier survival analysis, and Gene Set Enrichment Analysis (GSEA) were used to analyze the expression level, survival, and related mechanisms of SNORA38 in breast cancer. ISH and IHC were performed to analyze the correlation between SNORA38 and OCT-4, which showed that the expression of SNORA38 in breast cancer might be related to the stem cell regulation in BCSCs. Our results might provide theoretical support for finding a new diagnostic marker and therapeutic target for breast cancer.

## Materials and methods

### TCGA and Kaplan–Meier survival analysis

**Figure 1:**
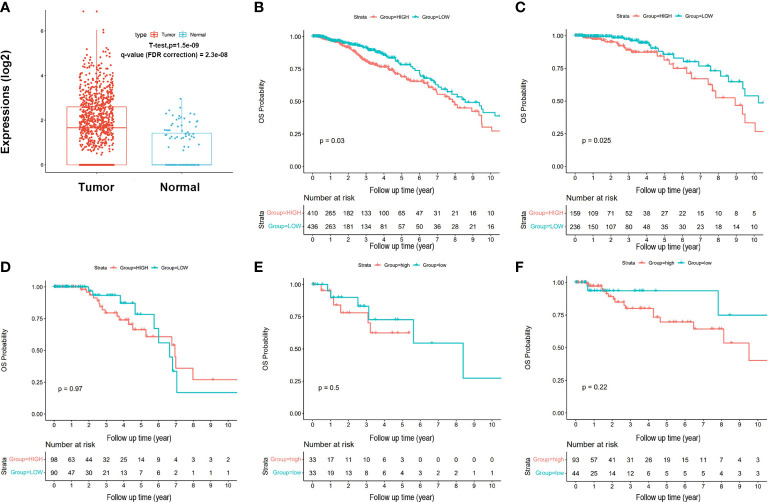
Expression and survival of SNORA38 signature in breast cancer. **(A)** SNORA38 expressed higher in breast cancer tissues than in normal tissues in TCGA (*P* < 0.05). **(B)** High SNORA38 expression was relevant to a shorter OS in all BC patients in Kaplan–Meier plotter (*P* = 0.030). **(C)** High SNORA38 expression was relevant to a shorter OS in luminal A patients in Kaplan–Meier plotter (*P* = 0.025). **(D)** High SNORA38 expression did not show any association with a shorter OS in luminal B patients in Kaplan–Meier plotter (*P* = 0.973). **(E)** High SNORA38 expression did not show any association with a shorter OS in Her-2 type patients in Kaplan–Meier plotter (*P* = 0.501). **(F)** High SNORA38 expression did not show any association with a shorter OS in basal-like type patients in Kaplan–Meier plotter (*P* = 0.225).

The TCGA-BRCA gene expression was downloaded from the database (https://cancergenome.nih.gov/), and 1,222 tissue samples were included, including 1,109 breast cancer tissue samples and 113 normal breast tissue samples. After integration and standardization of all data by using the edgR package, breast cancer tissue samples were divided into high and low expression groups according to the median value of the SNORA38 expression. “SNORic (snoRNA in cancers, http://bioinfo.life.hust.edu.cn/SNORic/basic/) was used to analyze the difference of SNORA38 expression between tumor group and normal group in breast cancer in TCGA-BRCA.” According to the website, the unit of the expression values is RPKM. The unit of ordinate shown in **Figure 1A** is log_2_. Overall survival (OS) in the total population of breast cancer patients and the correlation with OCT-4 and SNORA38 were analyzed by Graphpad Prism 8.0.

### Gene set enrichment analysis

The integrated and normalized data from TCGA-BRCA, classified by SNORA38 expression as we mentioned before and gene sets downloaded from the Molecular Signatures Database (MsigDB), were input to analyze the potential mechanism related to SNORA38 by GSEA. To ensure the credibility of the analysis results, we selected 1,000 permutations in the software. Pathways significantly associated with SNORA38 were screened based on normalized enrichment score (NES), while screening was based on normalized *P* < 0.05 and false discovery rate (FDR) < 25%.

### Breast cancer cell lines

MCF-10A and MCF-7 cell lines were obtained from the American Type Culture Collection (ATCC). Both cell lines were cultured by high-glucose (4.5 mg/ml) DMEM (Dulbecco’s modified eagle medium) (HyClone, Logan, Utah, USA) with 10% serum (Tianjin Hao Yang Biological Manufacture CL, China). MCF-7 CSCs were cultured by DMEM/F-12 (Gibco, Thermo Fisher Scientific) with 2% B27 (Gibco, Thermo Fisher Scientific, Waltham, USA) and EGF (epidermal growth factor) 20 ug/L (Promega, Shanghai, China). As previously reported (22), all cells used in the present study were cultured in 37°C, 5% CO_2_, and 95% air incubator.

SnoRNA chips (Oebiotech, Shanghai, China) were employed to investigate the differential snoRNA expression between BCCs (breast cancer cells) and BCSCs. In total, 248 snoRNAs were analyzed in this chip.

### Real-time quantitative polymerase chain reaction

Total RNA was isolated using TRIzol reagent (CWBIO, Beijing, China). RNA concentration was measured by NanoDrop 2000 spectrophotometer (Thermo Fisher Scientific, Waltham, USA). cDNA was obtained using the PrimeScript RT kit (TaKaRa, Japan). SYBR Premix Ex Taq I was used for RT-PCR. The mRNA internal control was β–actin, and U6 was internal control for snoRNA. 2^-ΔΔCt^method was used to calculate the relative RNA expression.

### Patients and tissue samples

Fixed and paraffin-embedded specimens (*n* = 77) and fresh cancer tissues with paired adjacent normal tissues (*n* = 16) were obtained with permission from the Ethics Review Committee of the First Affiliated Hospital of China Medical University (AF-SOP-07-1.1-01). Curative operations and reliable medical records were the basic principles of inclusion. Clinical and pathological information was obtained from the Hospital Information System, including data regarding age, tumor size, lymph nodes status, estrogen receptor (ER) status, progesterone receptor (PR) status, human epidermal growth factor, molecular subtypes, and Tumor Node Metastasis (TNM) stage.

### *In situ* hybridization

ISH was performed similarly to our previous study (23). In short, RNase enzymes were removed from all liquids and experimental devices. APES glue and DEPC-treated water were used to process the slides. The slides were dewaxed with xylene and rehydrated with gradient ethanol. Endogenous peroxidase activity was blocked by 3% hydrogen peroxide, and mRNA was exposed to pepsin. Digoxin-labeled oligonucleotide probes were incubated overnight at 37°C. According to snoRNAs ISH Kit (Boster) regimen, block solution, biotinylated rat anti digoxin, and SABC were added. Finally, DAB (3,3’-diaminobenzidine, DAB) staining was performed and assessed blind by two pathologists. A slide containing the target antigen was used as the positive control. Phosphate Buffered Saline (PBS) was used as the primary antibody in the negative control.

### Immunohistochemistry

IHC was performed similarly to our previous study (23). After conventional dewaxing and gradient ethanol dehydration, all sections were exposed to high pressure and citric acid buffer. Then, all sections were incubated overnight with anti-OCT4 (1:500, Cell Signaling Technology, USA) at 4°C. After rinsing with PBS, the antibody was incubated for 1 h with the secondary antibody at room temperature. Finally, sections were successively treated with DAB reagent and hematoxylin. A slide containing the target antigen was used as the positive control. PBS was used as the primary antibody in the negative control.

### Evaluation of ISH and IHC

DAB staining was assessed blind by two professional pathologists. SNORA38 and OCT-4 expression was assessed by staining intensity and percentage of positive staining cells. The percentage of positive cells were assigned as follows: 0 (< 5%), 1 (6–25%), 2 (26–50%), 3 (51–75%), and 4 (> 76%). The final ISH and IHC scores were multiplied by percentage score and intensity. All of the patients were divided into two groups: high SNORA38 expression (score ≥ 4) and low SNORA38 expression (score < 4).

### Statistical analysis

In the present study, statistical analyses were performed using SPSS 24.0 (Chicago, IL, USA) and Graphpad Prism 8.0 (La Jolla, CA, USA). The correlation between SNORA38 expression and clinical-pathological factors was measured by Pearson Chi-square test, Fisher’s exact test, and Logistic regression analysis. Univariate Cox regression analysis was conducted to assess the independent prognostic indicators for breast cancer patients, and then multivariate Cox regression analysis was performed within the characteristics selected in the univariate Cox regression analysis. Survival probabilities were measured by the Kaplan–Meier method and assessed by a log-rank test. OS curves were generated to evaluate the survival differences between the SNORA38-high and SNORA38-low patients. Receiver Operating Characteristic (ROC) analysis was used to analyze the diagnostic value, and the area under the ROC curve (AUC) greater than 0.5 was considered of diagnostic value. The relevance between SNORA38 and OCT-4 was calculated by Spearman correlation analysis. A *P*-value less than 0.05 was considered statistically significant.

## Results

### Expression of SNORA38 in breast cancer

**Figure 2:**
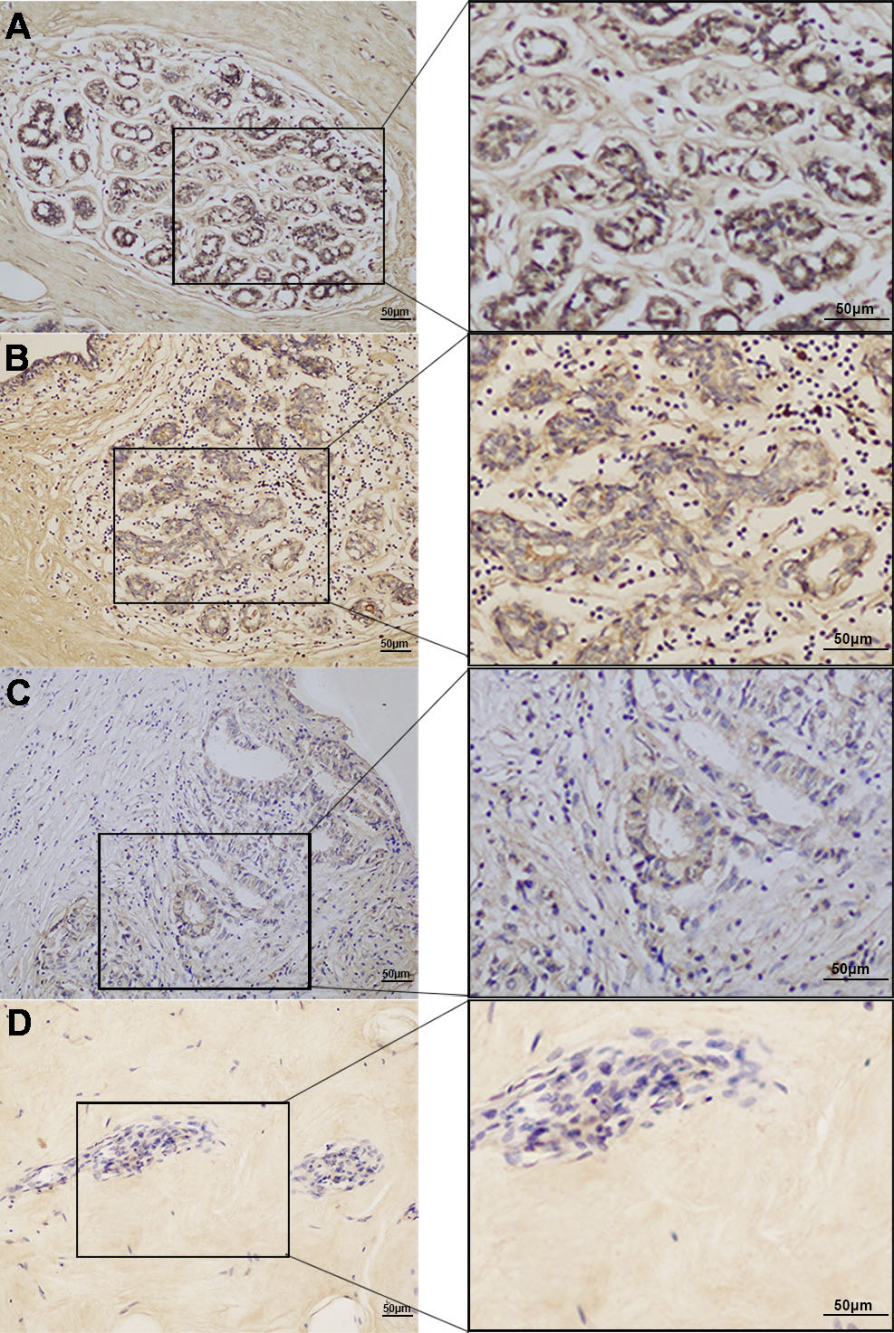
Expression of SNORA38 in breast cancer. **(A)** heavy stained, **(B)** medium stained, **(C)** light stained, and **(D)** no stained, respectively; original magnification, 200× (left) and 400× (right). Scale bars, 50 μm.

The expression of SNORA38 in TCGA showed that SNORA38 was up-regulated in breast cancer tissues compared with normal tissues. (*P* < 0.01, **Figure 1A**). To clarify whether SNORA38 is associated with breast cancer, SNORA38 expression levels in 77 paraffin-embedded samples were evaluated by ISH. Heavy stained (**Figure 2A**), medium stained (**Figure 2B**), light stained (**Figure 2C**), no stained (**Figure 2D**), as well as negative controls (**Figures S2A** and **S2C**) and positive controls (**Figures S2B** and **S2D**) were shown in **Figure 2** and **Supplement Figure S2**. The positive rate (33/77, 42.9%) of SNORA38 in breast cancer was significantly higher than that (3/16, 18.75%) in adjacent normal breast tissues (*P* < 0.05).

### Correlation between SNORA38 expression and clinical pathology factors

To further clarify how SNORA38 is involved in the development of breast cancer, the correlation between SNORA38 expression and clinicopathological factors was analyzed. Univariate analysis (**Table 1**) showed that SNORA38 expression was significantly correlated with tumor size (*P* = 0.01), lymph node metastasis (*P* = 0.006), ER (*P* = 0.024) and TNM stage (*P* = 0.040). Among these four variables (tumor size, lymph node metastasis, ER, and TNM stage), only tumor size was a significant predictive factor for SNORA38 expression in the multivariate analysis (**Table 2**).

**Table 1:** Univariate analysis of SNORA38 expression and clinical pathology factor.

Factors	Number	SNORA38 expression		*x* ^2^	*P*	Crude OR (95% CI)
	(%)	High (%)	Low (%)			
Age (years)				2.818	0.093^a^	
≤ 60	58 (75.3)	28 (73.7)	30 (26.3)		0.067^b^	2.897 (0.929 - 9.035)
> 61	19 (24.7)	5 (26.3)	14 (73.7)			Reference
Tumor size				6.676	0.010^a^	
≥ 3 cm	43 (55.8)	24 (55.8)	19 (44.2)		0.011^a^	3.509 (1.329 - 9.265)
< 3 cm	34 (44.2)	9 (26.5)	25 (73.5)			Reference
LN Metastases					0.006^a^	
Negative	53 (66.8)	17 (32.1)	36 (67.9)	8.071		Reference
Positive	24 (31.2)	16 (66.7)	8 (33.3)		0.006^b^	4.235 (1.518 - 11.818)
ER				5.096	0.024^a^	
Negative	22 (28.6)	5 (22.7)	17 (77.3)			
Positive	55 (71.4)	28 (50.9)	27 (49.1)		0.029^b^	3.526 (1.141 - 10.900)
PR				1.540	0.215^a^	
Negative	27 (35.1)	9 (33.3)	18 (66.7)			Reference
Positive	50 (64.9)	24 (48.0)	26 (52.0)		0.217^b^	1.846 (0.697 - 4.888)
Her-2				0.461	0.497^a^	
Negative	48 (62.3)	22 (45.8)	26 (54.2)			Reference
Positive	29 (37.7)	11 (37.9)	18 (62.1)		0.498^b^	0.7222 (0.282 - 1.850)
Molecular typing				6.844	0.077^a^	
Luminal A	20 (26.0)	12 (60.0)	8 (40.0)		0.035^b^	6.750 (1.145 - 39.796)
Luminal B	37 (48.0)	17 (45.9)	20 (54.1)		0.114^b^	3.825 (0.725 - 20.177)
Her-2	9 (11.7)	2 (22.2)	7 (77.8)		0.822^b^	1.286 (0.143 - 11.543)
TNBC	11 (14.3)	2 (18.2)	9 (81.8)			Reference
TNM staging					0.040^a^	
I	25 (32.5)	8 (32.0)	17 (68.0)	6.461		Reference
II	45 (58.4)	19 (42.2)	26 (57.8)		0.401^b^	1.553 (0.556 - 4.340)
III	7 (91.0)	6 (85.7)	1 (14.3)		0.028^b^	12.75 (1.307 - 124.3)

p-value a came from Pearson chi-square tests or Fisher’s Exact Test P-value b came from logistic regression analyses.

**Table 2:** Multivariate analysis of SNORA38 expression and clinical pathology factor.

Factors	Number	SNORA38 expression		*P*	Crude OR (95 CI)
	(%)	High (%)	Low (%)		
Tumor size					
≥ 3 cm	43 (55.8)	24 (55.8)	19 (44.2)	0.022	4.669 (1.249 - 17.448)
< 3 cm	34 (44.2)	9 (26.5)	25 (73.5)		Reference
LN Metastases					
Negative	53 (68.8)	17 (32.1)	36 (67.9)		Reference
Positive	24 (31.2)	16 (66.7)	8 (33.3)	0.057	3.591 (0.962 - 13.411)
ER					
Negative	22 (28.6)	5 (22.7)	17 (77.3)		Reference
Positive	55 (71.4)	28 (50.9)	27 (49.1)	0.065	3.374 (0.926 - 12.295)
TNM staging					
I	25 (32.5)	8 (32.0)	17 (68.0)		Reference
II	45 (58.4)	19 (42.2)	26 (57.8)	0.199	2.656 (0.599 - 11.782)
III	7 (9.1)	6 (85.7)	1 (14.3)	0.667	0.547 (0.035 - 8.517)

### Correlation between SNORA38 and prognosis in breast cancer patient

Kaplan–Meier survival analysis and the log-rank test showed that SNORA38 was significantly correlated with shorter OS in breast cancer patients (*P* = 0.03, **Figure 1B**). Higher SNORA38 expression was significantly correlated with shorter OS in luminal A breast cancer patients (*P* = 0.025, **Figure 1C**); however, there was no obvious correlation between OS and SNORA38 among other types of breast cancer patients: luminal B patients (*P* = 0.97, **Figure 1D**), Her-2 type patients (*P*= 0.50, **Figure 1E**), and basal-like-type patients (*P* = 0.22, **Figure 1F**). Then, we used univariate Cox regression analysis to evaluate the influence of clinical-pathological factors on OS in breast cancer patients. OS were significantly correlated with age (*p* = 0.017), lymph node metastasis (*P* = 0.028), and SNORA38 expression (*P* = 0.031) (**Supplementary Table S1**). In addition, multivariate Cox regression analysis showed that age (*p* = 0.013), lymph node metastasis (*P* = 0.046), and SNORA38 expression (*p* = 0.030) were prognostic factors for OS shortening in breast cancer patients. To evaluate whether SNORA38 expression can be used as a predictor of breast cancer, ROC was used for analysis, which showed that the AUC of SNORA38 was 0.563.

### SNORA38-related signaling pathways in GSEA

**Figure 3:**
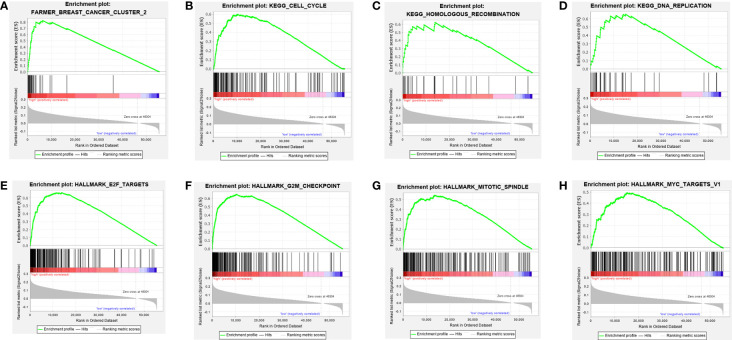
Enrichment plots from Gene Set Enrichment Analysis (GSEA). GSEA was used to indicate the mechanisms related to SNORA38 expression in breast cancer. GSEA disclosed a significant enrichment of **(A)** Breast cancer cluster 2. **(B)** Cell_cycle. **(C)** DNA_replication. **(D)** Homologous recombination. **(E)** E2F targets. **(F)** G2M checkpoint. **(G)** MITOTIC spindle. **(H)** MYC targets.

According to NES, significantly enriched pathways were associated with the biological processes of breast cancer (**Figures 3A–H** and **Table 3**). TCGA-BRCA samples in the group with high expression of SNORA38 were enriched in cell cycle, DNA replication, homologous recombination, E2F targets, G2M checkpoint, mitotic spindle, myc targets, and so forth.

**Table 3:** Gene set enriched with SNORA38 high expression.

MsigDB collection	Gene set name	NES	NOM *p*-val	FDR *q*-val
c2.cgp.v6.2.symbols.gmt	FARMER_BREAST_CANCER_CLUSTER_2	2.270	0.000	0.000
c2.cp.kegg.v6.2.symbols.gmt	KEGG_CELL_CYCLE	1.819	0.003	0.002
	KEGG_DNA_REPLICATION	1.796	0.003	0.001
	KEGG_HOMOLOGOUS_RECOMBINATION	1.688	0.027	0.007
h.all.v6.0.symbols.gmt	HALLMARK_E2F_TARGETS	2.060	0.000	0.000
	HALLMARK_G2M_CHECKPOINT	1.998	0.000	0.000
	HALLMARK_MITOTIC_SPINDLE	1.674	0.000	0.000
	HALLMARK_MYC_TARGETS_V1	1.532	0.000	0.002

NES, normalized enrichment score; NOM, nominal; FDR, false discovery rate.

### SNORA38 expressed higher in the BCSCs than BCCs and was related to breast cancer stemness

**Figure 4:**
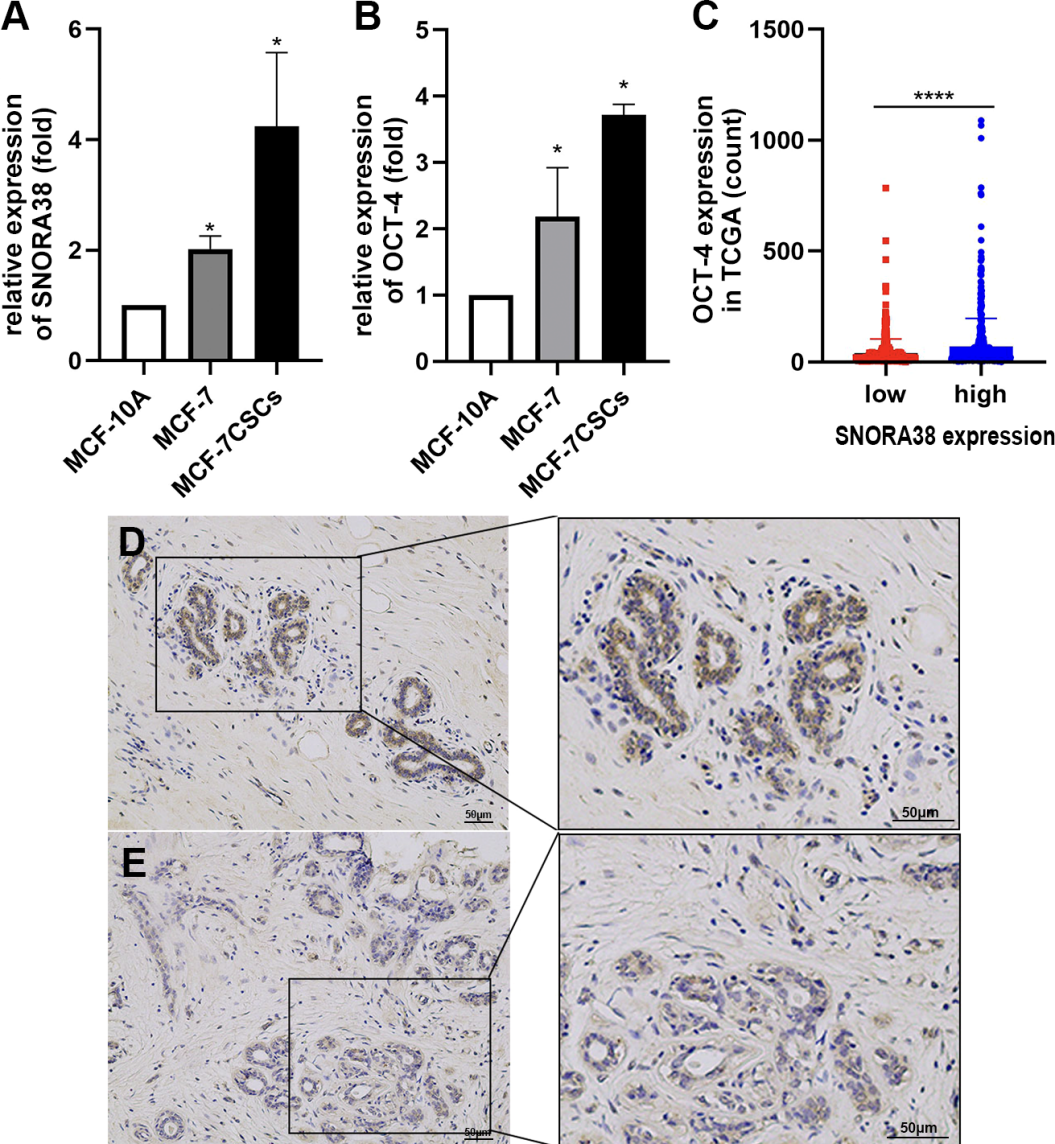
SNORA38 expressed higher in the BCSCs than BCCs and was related to breast cancer stemness. **(A)** relative expression of SNORA38 in MCF-10A,MCF-7, and MCF-7CSCs **(B)** relative expression of OCT-4 in MCF-10A, MCF-7, and MCF-7CSCs **(C)** OCT-4 expressed higher in high SNORA38 expression group than in low SNORA38 expression group in breast cancer in TCGA **(D)** high expression of OCT-4 in IHC **(E)** low expression of OCT-4 in IHC *P < 0.05 and ****P < 0.0001.

The induction and culture of BCSCs were matured (24). After 7–8 days of culture, MCF-7 CSCs possessed typical phenotypic characteristics, such as high CD44^+^CD24^−^ phenotype and high expression of CSCs markers (OCT-4) at mRNA levels (**Figure 4A**). snoRNA chips (Oebiotech, Shanghai, China) included 248 snoRNAs that were employed to investigate the differential snoRNAs expression between BCCs (breast cancer cells) and BCSCs, which revealed that SNORA38 was significantly overexpressed in CD44^+^CD24^−^ subgroup (**Figure S1**). The results of microarray analysis were further validated by real-time qPCR, which showed that the expression of SNORA38 in MCF-7 CSCs was higher than that in MCF-7 (**Figure 4B**). In addition, OCT-4 was correlated with SNORA38 in TCGA (*P* = 6.2e^-07^, *q*-value (FDR correction) = 1.24e^-07^), as shown in **Figure 4C**, and a positive correlation was also observed between SNORA38 (through ISH) and OCT-4 (through IHC, **Figures 4D, E** and **Supplementary Figure S2**) as shown in **Table 4**. The above results further suggested that SNORA38 is associated with carcinogenic characteristics and increased stem phenotype.

**Table 4:** Correlation analysis of SNORA38 expression and OCT-4.

Factors	Number	SNORA38 expression		Spearman	*p*-value
	(%)	High (%)	Low (%)	rs	
OCT-4 expression					
High (%)	35 (45.5)	20 (57.1)	15 (42.9)	0.264	0.021
Low (%)	42 (54.5)	13 (30.1)	29 (69.9)		

## Discussion

snoRNAs are a class of non-coding RNAs that play critical regulatory roles in various physiological and pathological events (24–26). Previous studies have shown that some snoRNAs can participate in the regulation of the metastasis and recurrence of breast cancer (17, 27). In addition, snoRNAs are related to the therapeutic resistance of cancer (28–32). Thus, snoRNAs are recognized as diagnostic and prognostic indicators and as therapeutic targets in cancer. Like protein-coding genes and miRNAs, snoRNAs have oncogenic and tumor-suppressive functions (27, 33). snoRNAs regulate the expression of protein-coding genes through different mechanisms. Some computational studies predicted that snoRNAs can interact with other RNA types to regulate biological function and cellular signaling pathways (34). Several studies demonstrated the association between abnormal snoRNAs expression and breast cancer progression (35, 36). We analyzed the differential expression of snoRNAs by snoRNAs chips and found that SNORA38 was significantly up-regulated in BCSCs. However, there have been no reports of SNORA38 expression in breast cancer so far. Therefore, the present study was carried out to explore the potential significance of SNORA38 in breast cancer.

According to TCGA analyses and experimental evidence, SNORA38 was highly expressed in breast cancer tissues and BCSCs, suggesting that overexpression of SNORA38 may promote malignant transformation and play an essential role in the genesis and development of breast cancer. A recent study showed that snoRNAs were associated with clinical-pathological factors, including lymphatic invasion and distant metastasis (15). SNORA38 expression was correlated with tumor size, lymph node metastasis, TNM stage, and ER expression in univariate analysis. Therefore, these results suggested that the overexpression of SNORA38 might predict breast cancer cells proliferation and invasion. However, multivariate analysis did not prove a statistical significance in lymph node metastasis, which might be due to the insufficient sample size.

According to a recent study, low expression of snoRA52 is related to poor long-term survival in hepatocellular carcinoma (37). The relationship between SNORA38 expression and prognosis in breast cancer patients has not been discussed. The present study found that SNORA38 was a prognostic factor in breast cancer. Higher SNORA38 expression indicated a shorter OS in the whole breast cancer population and luminal A breast cancer patients. Previous studies have shown that compared with miRNA, ctDNA (circulating tumor DNA), and exosome, snoRNAs were more stable, technically easy to enrich and detect, therefore, more suitable to serve as prognostic biomarkers (38–41). In malignant tumors, snoRNAs are associated with clinicopathological features and disease status. snoRNAs also have a specific distribution in plasma and other body fluids; therefore, they can be used as potential biomarkers of liquid biopsy (42). The above studies suggested that snoRNAs might be new prognostic markers. Previous studies have reported that aberrant methylation of genes leads to breast cancer. DNA methylation occurs before protein translation, which might have greater value in the early diagnosis of breast cancer than the detection of cancer-related protein expression. Although individual gene methylation showed good specificity for breast cancer, it was often less sensitive for breast cancer diagnosis alone, so it was necessary to jointly detect changes in multiple gene methylation sites and construct a detailed methylation map (43).

Similarly, we recognized the limitations of snoRNA alone as a prognostic marker. Therefore, we look forward to snoRNAs combined with other present prognostic markers based on gene expression or DNA methylation in the future to obtain a better prognostic effect. Moreover, the ROC analysis of SNORA38 did not show statistical significance, which may be due to several reasons. First, the number of samples included in this study is relatively small. Second, breast cancer cells are highly heterogeneous, and the mechanism of snoRNAs in tumor genesis and development is so complex that a certain snoRNA may participate in various pathways and have multiple biological functions in different types of cells (44, 45). Therefore, expanding the sample size, exploring the mechanism of multiple indicators, and analyzing the prognostic value in different types of breast cancer cells may advance the field.

The occurrence and development of malignant tumors may involve multiple signaling pathways (46). The present study identified the underlying mechanisms by which SNORA38 might influence the occurrence and development of breast cancer. From GSEA, SNORA38 was involved in several signaling pathways and may be associated with tumor cell survival and proliferation by influencing the cell cycle, DNA replication, homologous recombination, and mitotic spindle. SNORA38 was associated with E2F targets and G2M checkpoint, suggesting that SNORA38 may be involved in cell apoptosis (47, 48). SNORA38 was closely association with MYC targets, suggesting that SNORA38 may be involved in tumor cell cycle and related to apoptosis and cell transformation (49). These results provided a new idea for understanding the molecular mechanism of SNORA38, regulating the biological process of malignant tumors. Because the molecular function of SNORA38 has not been fully studied, further studies are needed to clarify its role in tumor genesis and metastasis.

To sum up, we considered SNORA38 a potential diagnostic marker and therapeutic target for several reasons. First, the expression of SNORA38 in breast cancer tissues was higher than that in normal tissues, which was significantly correlated with tumor size, lymph node metastasis, and TNM stage, suggesting that SNORA38 may be related to the occurrence and development of breast cancer. Second, breast cancer patients with high expression of SNORA38 had a poor prognosis that might be a prerequisite for SNORA38 to become a therapeutic target. Third, SNORA38 was associated with OCT-4, a recognized stem cell marker that regulates BCSCs’ self-renewal, which indicated that SNORA38 might cause tumors by targeting BCSCs. BCSCs are characterized by self-renewal and pluripotency, which were thought to be the source of drug resistance and relapse of breast cancer (7, 8). Therefore, the intervention of stem cells and the targeting of signaling pathways have shown promising prospects in breast cancer therapy.

## Conclusions

In conclusion, SNORA38 was significantly up-regulated in breast cancer tissues compared with normal tissues. The higher expression of SNORA38 was related to larger tumor size, more lymph node metastasis, higher TNM staging, and shorter OS in breast cancer. The correlation between SNORA38 and stem cell marker OCT-4 and the potential mechanism associated with tumor cell survival and proliferation suggested that SNORA38 might be related to the stemness of breast cancer cells and might be a potential therapeutic target in breast cancer in the future.

## Data availability statement

The original contributions presented in the study are included in the article/**Supplementary Material**. Further inquiries can be directed to the corresponding authors.

## Ethics statement

Written informed consent was obtained from the individual(s) for the publication of any potentially identifiable images or data included in this article.

## Author contributions

FJ and AZ conceived and supervised the study. JS, SL, and WZ designed and performed the experiments. ZJ and XL collected clinical data and performed data statistics. ZJ and JS revised the manuscript. MZ performed the experiments. All authors contributed to the article and approved the submitted version.

## Funding

This study was financially supported by China Postdoctoral Science Foundation (Grant Number: 258078), National Natural Science Foundation of China (Grant Number: 82073282), and Natural Science Foundation of Liaoning Province - Doctoral Research Program (Grant Number:2021-BS-1115).

## Conflict of interest

The authors declare that the research was conducted in the absence of any commercial or financial relationships that could be construed as a potential conflict of interest.

## Publisher’s note

All claims expressed in this article are solely those of the authors and do not necessarily represent those of their affiliated organizations, or those of the publisher, the editors and the reviewers. Any product that may be evaluated in this article, or claim that may be made by its manufacturer, is not guaranteed or endorsed by the publisher.

## Supplementary material

Supplementary Figure 1 Heat maps of differential snoRNAs between CD44^+^ CD24^−^ subgroup and CD44^−^ CD24^+^ subgroup in microarray expression profile.

Supplementary Figure 2 Positive and negative staining in ISH and IHC. (**A**) negative staining of ISH (**B**) positive staining of ISH (**C**) negative staining of IHC (**D**) positive staining of IHC. Original magnification, 200× (left) and 400× (right). Scale bars, 50 μm.
