# Supplementary figures and images for: Clinical significance and prognostic value of small nucleolar RNA SNORA38 in breast cancer
Source: Front Oncol. 2022 Sep 9;12:930024. doi: 10.3389/fonc.2022.930024 (PMC9500313; doi:10.3389/fonc.2022.930024)

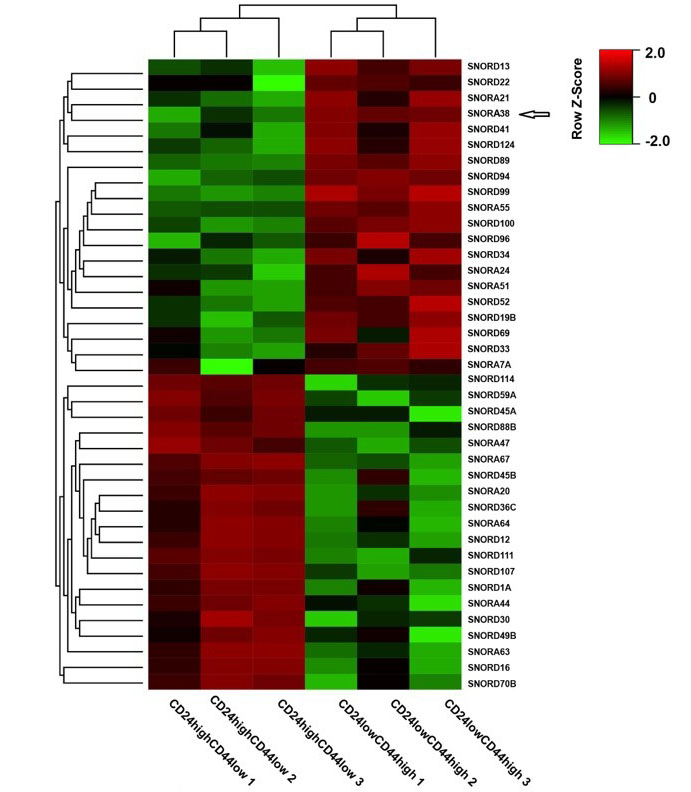

Supplement: Supplementary Figure 1 — Heat maps of differential snoRNAs between CD44+ CD24− subgroup and CD44− CD24+ subgroup in microarray expression profile. [file Image_1.jpg]

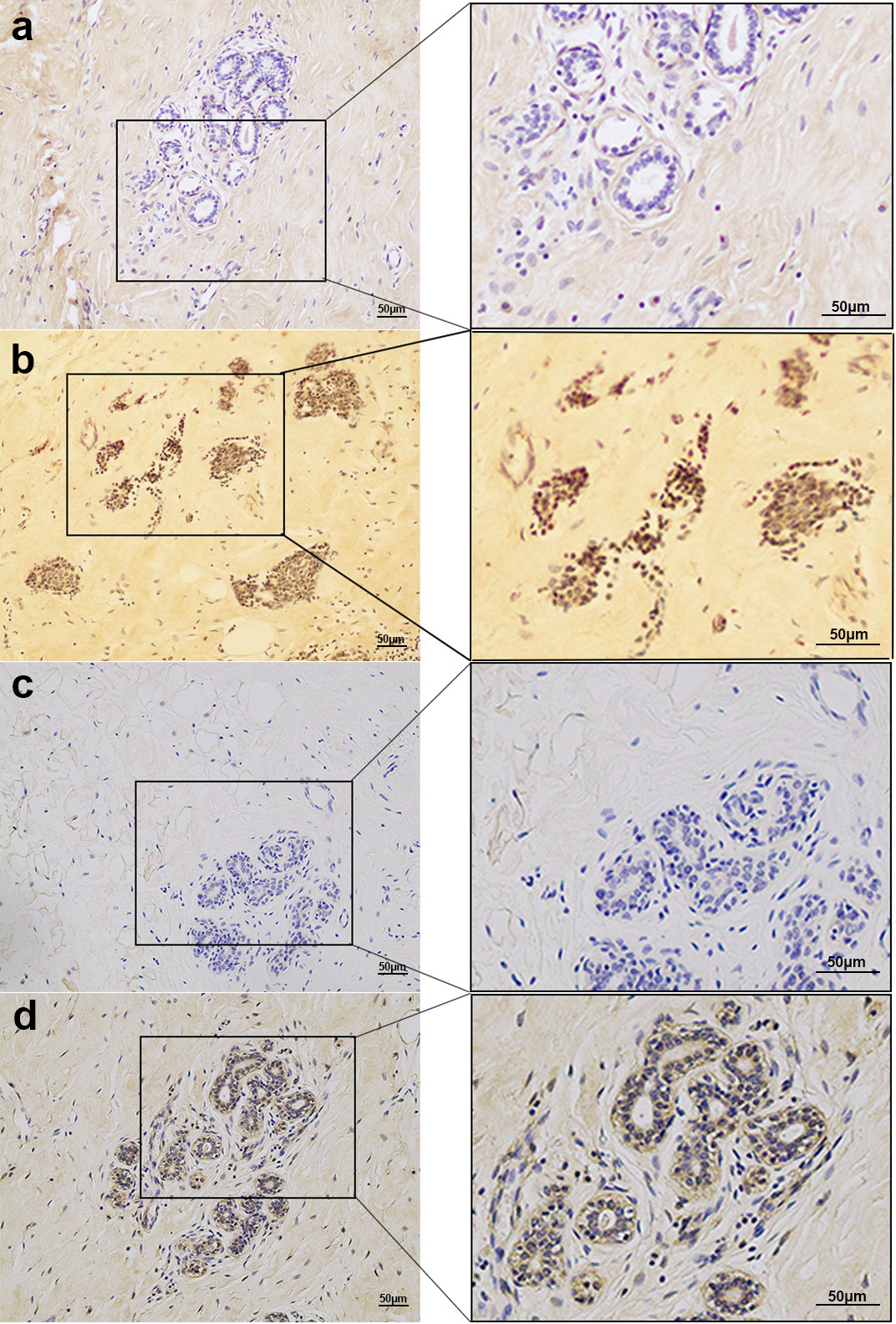

Supplement: Supplementary Figure 2 — Positive and negative staining in ISH and IHC. (A) negative staining of ISH (B) positive staining of ISH (C) negative staining of IHC (D) positive staining of IHC. Original magnification, 200× (left) and 400× (right). Scale bars, 50 μm. [file Image_2.tif]
