# Supplementary material for: Clinical significance and prognostic value of small nucleolar RNA SNORA38 in breast cancer
Source: Front Oncol. 2022 Sep 9;12:930024. doi: 10.3389/fonc.2022.930024 (PMC9500313; doi:10.3389/fonc.2022.930024)
Supplement: Supplementary file 3 [file Table_1.docx]

| **Table S1. Univariable and** **multivariable analysis of** **overall survival** | | | | | | | |
| --- | --- | --- | --- | --- | --- | --- | --- |
| **Factors** | **Univariate Analysis** | | | **Multivariate Analysis** | | | |
|  | **P** | **HR** | **95 CI** | **P** | **Adjusted HR** | **95 CI** |  |
| **Age(years)** |  |  |  |  |  |  |  |
| **≤ 60** | 0.017 | 1.589 | 1.086-2.323 | 0.013 | 1.673 | 1.116 – 2.507 |  |
| **> 60** | Reference |  |  | Reference |  |  |  |
| **Tumor Size** |  |  |  |  |  |  |  |
| **≥ 3 cm** | 0.332 | 1.271 | 0.782-2.066 |  |  |  |  |
| **< 3 cm** | Reference |  |  |  |  |  |  |
| **LN Metastases** |  |  |  |  |  |  |  |
| **negative** | Reference |  |  | Reference |  |  |  |
| **positive** | 0.028 | 1.590 | 1.051-2.406 | 0.046 | 1.532 | 1.007 - 2.331 |  |
| **ER** |  |  |  |  |  |  |  |
| **negative** | Reference |  |  |  |  |  |  |
| **positive** | 0.899 | 0.570 | 0.531-1.417 |  |  |  |  |
| **PR** |  |  |  |  |  |  |  |
| **negative** | Reference |  |  |  |  |  |  |
| **positive** | 0.181 | 0.757 | 0.503-1.138 |  |  |  |  |
| **Her2** |  |  |  |  |  |  |  |
| **negative** | Reference |  |  |  |  |  |  |
| **positive** | 0.697 | 1.129 | 0.613-2.082 |  |  |  |  |
| **SNORNA38** |  |  |  |  |  |  |  |
| **High Expression** | 0.031 | 1.457 | 1.034-2.054 | 0.030 | 1.583 | 1.047-2.395 |  |
| **Low Expression** | Reference |  |  | Reference |  |  |  |
| **Molecular typing** |  |  |  |  |  |  |  |
| **Luminal A** | 0.037 | 0.461 | 0.223-0.956 |  |  |  |  |
| **Luminal B** | 0.004 | 0.391 | 0.206-0.744 |  |  |  |  |
| **Her-2** | 0.528 | 0.805 | 0.410-1.580 |  |  |  |  |
| **TNBC** | Reference |  |  |  |  |  |  |
| **TNM staging** |  |  |  |  |  |  |  |
| **I** | Reference |  |  |  |  |  |  |
| **II** | 0.259 | 1.490 | 0.746-2.978 |  |  |  |  |
| **III** | 0.001 | 3.513 | 1.630-7.570 |  |  |  |  |
